# Is Intramural Hematoma a Complication of COVID-19 Disease?

**DOI:** 10.1055/s-0041-1724006

**Published:** 2021-10-04

**Authors:** Makoto Mori, Arnar Geirsson, Prashanth Vallabhajosyula, Roland Assi

**Affiliations:** 1 Division of Cardiac Surgery, Yale University School of Medicine, New Haven, Connecticut

We read the report by Terzi and colleagues 1 from Italy with great interest regarding a patient with novel coronavirus disease 2019 (COVID-19) who presented with intramural hematoma. As we congratulate the team for the successful management of this complex patient, we felt that the claimed association between the cytokine storm and aortic wall injury may be unsubstantiated. The article title went as far to suggest that intramural hematoma was a complication of COVID-19. Although this speculated association is interesting and worth further examination, this case report alone neither supports or refutes this association which may simply be a coincidence.

Although likely multifactorial, the number of cases presented for acute Type A dissection in New York declined during the pandemic, 2 which is an observation that may contradict with the claim that COVID-19 may cause intramural hematoma. Additionally, in the absence of the description of the family history of aortic disease or the pathological examination results, we cannot judge whether this was indeed related to COVID-19. We recently encountered a 52-year-old man with COVID-19 with intramural hematoma, who had a family history of aortic aneurysm and underwent emergent operation. 3 He was otherwise healthy but had a history of respiratory illness several weeks prior to the presentation, although he was without respiratory symptoms at the time of presentation. In the operating room, we did not note any gross differences in the appearance of the intraluminal or extraluminal ascending aorta or the arch. Our case underwent pathological examination which reported myxomatous degeneration of the aortic wall, similar to aneurysm and intramural hematoma in non-COVID-19 patients. The report also noted the presence of chronic inflammation which may be an unusual finding but of unclear significance. The specimens were not tested for severe acute respiratory syndrome-coronavirus-2 (SARS-CoV-2).

The case is nonetheless interesting, as there is a well-recognized entity of inflammatory aortic aneurysms, for which the etiology is chronic inflammatory conditions of various causes. 4 Additionally, there are potential aortic manifestation of this disease, such as luminal thrombus. 3 It would be interesting to study intraoperative gross appearances of such patients with aortic disease and history of COVID-19, as inflammatory aortic aneurysm is typically characterized by marked thickening of the aortic wall and fibrosis with adhesions to the surrounding tissue. 4
